# Dual adversarial models with cross-coordination consistency constraint for domain adaption in brain tumor segmentation

**DOI:** 10.3389/fnins.2023.1043533

**Published:** 2023-04-13

**Authors:** Chuanbo Qin, Wanying Li, Bin Zheng, Junying Zeng, Shufen Liang, Xiuping Zhang, Wenguang Zhang

**Affiliations:** ^1^Faculty of Intelligent Manufacturing, Wuyi University, Jiangmen, China; ^2^Department of Neurosurgery, Jiangmen Central Hospital, Jiangmen, China

**Keywords:** dual student, adversarial learning, semi-supervision, unsupervised domain adaptation, brain tumor, MR image

## Abstract

The brain tumor segmentation task with different domains remains a major challenge because tumors of different grades and severities may show different distributions, limiting the ability of a single segmentation model to label such tumors. Semi-supervised models (e.g., mean teacher) are strong unsupervised domain-adaptation learners. However, one of the main drawbacks of using a mean teacher is that given a large number of iterations, the teacher model weights converge to those of the student model, and any biased and unstable predictions are carried over to the student. In this article, we proposed a novel unsupervised domain-adaptation framework for the brain tumor segmentation task, which uses dual student and adversarial training techniques to effectively tackle domain shift with MR images. In this study, the adversarial strategy and consistency constraint for each student can align the feature representation on the source and target domains. Furthermore, we introduced the cross-coordination constraint for the target domain data to constrain the models to produce more confident predictions. We validated our framework on the cross-subtype and cross-modality tasks in brain tumor segmentation and achieved better performance than the current unsupervised domain-adaptation and semi-supervised frameworks.

## 1. Introduction

Glioma, a tumor originating from glial cells, is one of the most common primary brain tumors. Accurate automatic brain segmentation is key to the accurate delineation of brain tumor regions on neuroimaging, which is required to formulate useful clinical practice guidelines and understand the disease and its clinical challenges. However, despite the tireless efforts of researchers, accurate automatic brain tumor segmentation on medical images has remained a technical challenge due to domain heterogeneity issues, domain shifts, costly and time-consuming labeling, low-contrast imaging, and data imbalance.

**Figure 1:**
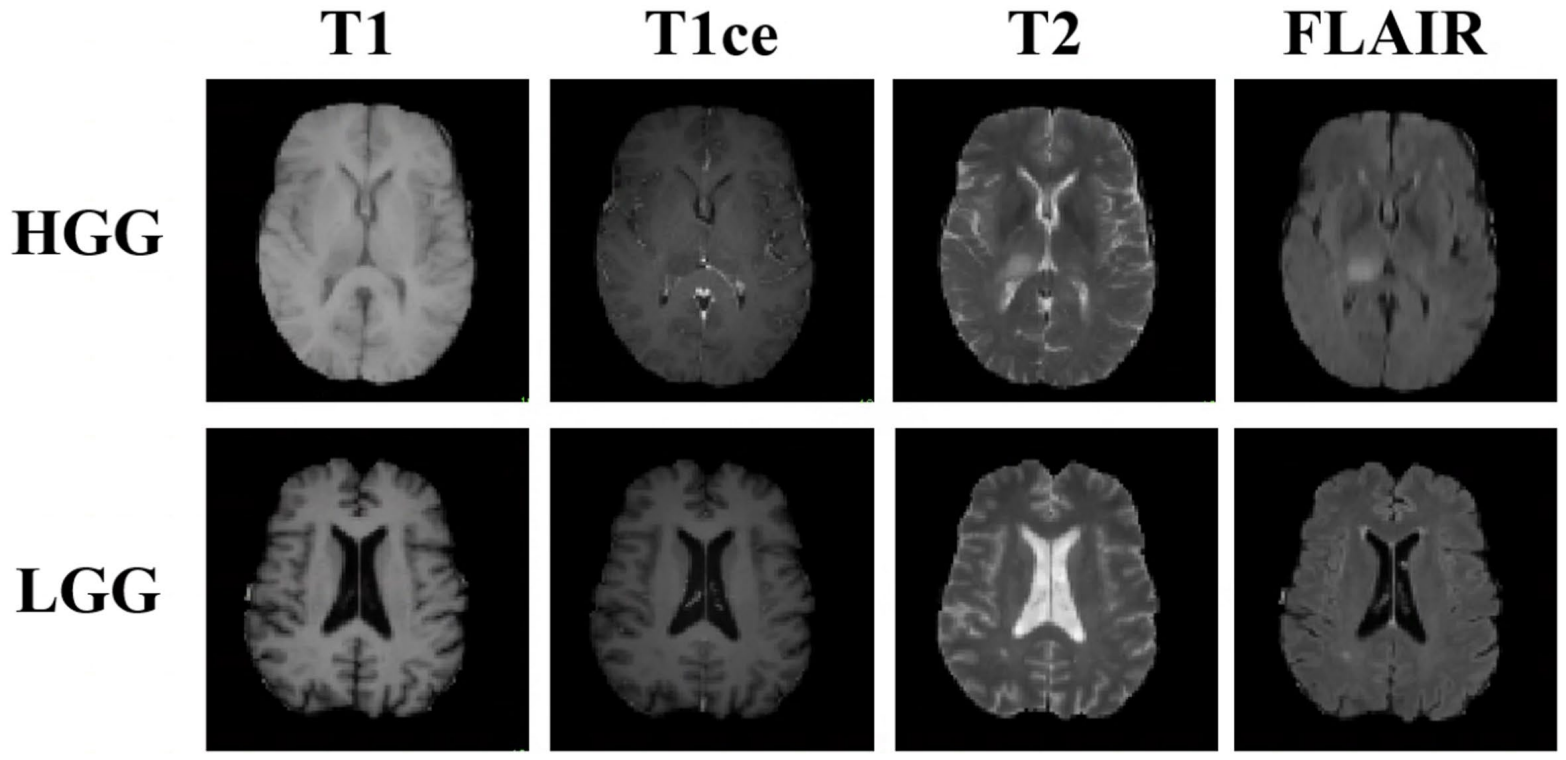
Examples of three-dimensional brain MR images of low-grade glioma (LGG) and high-grade glioma (HGG) samples. Each sample has images from four modalities: T1-weighted MRI, contrast-enhanced T1-weighted (T1ce) MRI, T2-weighted MRI, and fluid attenuation inversion recovery (FLAIR) MRI. These sequences provide complementary information for different subregions of brain tumors.

With the promising progress made in medical image-segmentation models (Myronenko, 2019; Li et al., 2021; Hatamizadeh et al., 2022; Luu and Park, 2022), several deep learning methods have been applied to automatically extract feature representations, and stable performance has been achieved in the test set of the experimental environment. However, in practice, the segmentation results are not always as expected because images acquired in different institutions can differ in terms of the image-acquisition parameters as well as the tumor distribution, grade, and severity (Figure 1). These differences can limit the learning ability of segmentation models that are trained using images of both high-grade gliomas (HGGs) and low-grade gliomas (LGGs). Furthermore, in model training using multimodal images, cross-modality domain shifts may arise (for example, a shift from T2-weighted images to T1-weighted images), leading to considerable performance degradation. In such cases, unsupervised domain adaptation (UDA) is useful for brain tumor segmentation as it can enable convolutional neural networks to extensively study existing labeled images from multiple modalities as a source-domain and unlabeled images from the target domain. Promising results have been achieved in the context of semantic segmentation by using domain-invariant feature training with a self-ensembling technique for MRI domain adaptation (Sener et al., 2016), primarily utilizing both source and target domains belonging to the same modality, self-supervised and adversarial training (Li et al., 2022), and generative models (Hassan et al., 2018).

Numerous UDA methods have been proposed in the literature, with a growing emphasis on learning to map representations of the source domain to those of the target domain while minimizing the distribution discrepancy (Long et al., 2016). In addition, many adversarial learning methods train domain-classifier networks (Sun et al., 2015; Sun and Saenko, 2016; Purushotham et al., 2017) to distinguish features as either a source or a target and train a feature-generator network to mimic the discriminator. In contrast, Ganin et al. (2016) employed domain-adversarial training and achieved state-of-the-art domain-adaptation performance on two distinct classification problems (document sentiment analysis and image classification). This technique validated adversarial learning and possesses great potential in domain-shift problems. Furthermore, several semi-supervised learning (SSL) methods have been employed on UDA tasks (Ding et al., 2018; Zou et al., 2018, 2019; Zhang et al., 2020) because SSL is a special case of UDA problems (Zhang et al., 2021), and SSL methods are typically motivated by basic assumptions about the data structure, such as the smoothness assumption. For example, self-ensembling is a variant of the mean teacher (Tarvainen and Valpola, 2017), and minimum class confusion shares similar objectives to entropy minimization and self-training. The superior results obtained also imply the efficacy of SSL methods for UDA tasks. Despite such progress, domain adaptation still needs to be explored in detail, and promising results may be achieved by developing SSL frameworks.

For medical image segmentation, Shanis et al. (2019) focused on brain tumor intramodality domain adaptation using self-ensembling and adversarial training. The authors demonstrated the effectiveness of the mean-teacher network and adversarial loss for UDA on medical datasets. Current unsupervised learning methods are directed toward combining multiple techniques to achieve superior performance in domain-adaptation tasks (Saito et al., 2018; Perone et al., 2019). This inspired us to further explore the performance of different SSL methods on UDA tasks. In this study, we address two domain-adaptation problems: one where the source and target domains belong to the same modality but contain tumors of different grades, and another where these domains belong to different imaging modalities. Multimodal image information complements each other, which improves the accuracy of segmentation but also increases the difficulty of the segmentation process to a certain extent. The use of multimodal images increases the information available for segmentation but simultaneously adds a large amount of unnecessary information, thereby limiting the learning effectiveness of the segmentation model. These cases are often neglected in biomedical image analysis as most deep learning networks are trained and tested on a mixture of data collected from different institutions and devices, yielding unpredictable performance if the test set is from a data source different from the training set.

In this study, we propose a combined framework that uses discriminators for aligning feature spaces, namely dual student models to break the limits of the consistency constraint instead of coupling the weights. We demonstrate the performance of our method on the Brain Tumor Segmentation 2019 (BraTS 2019) dataset. Specifically, we applied our method to perform the following two tasks:

1. Cross-subtype task: We used images of HGGs as the source domain and those of LGGs as the target domain for HGG-to-LGG domain adaptation.
2. Cross-modality task: Because the whole tumor was annotated using T2-weighted images in clinical practice, the tumor region and peritumoral edema were highlighted on fluid-attenuated inversion recovery (FLAIR) images and T2-weighted images, and the core tumor region without peritumoral edema was more visible on T1-weighted and contrast-enhanced T1-weighted (T1ce) images. We used the T2-weighted and FLAIR images as the labeled source domain and the T1-weighted and T1ce images as the target domain, which provided complementary information for the different subregions of brain tumors and a larger domain shift than that present in the cross-subtype task.

To the best of our knowledge, our study is the first to use the combination of a dual student model and adversarial learning for brain tumor domain adaptation. Furthermore, we did not need an additional source-domain network or class-ratio priors (Vu et al., 2019) based on the distribution of classes over the source labels.

## 2. Related work

### 2.1. Unsupervised domain adaptation

Unsupervised domain adaptation has become an important technique to alleviate the problem of highly variable data sources and costly labeling in a new domain because UDA does not rely on labeled training samples from the desired target domain. For the segmentation of white-matter hyperintensities, Orbes-Arteaga et al. (2019) proposed using a paired consistency loss to guide the adaptation and supplementing this with adversarial loss to prevent the model from being trapped in bad local minima. Deep co-training with the source domain and target domain is a conventional domain-adaptation training strategy. Due to the limited source-domain data and privacy issues, Liu et al. (2021) applied source-free UDA for segmentation, which used a pre-trained model rather than the conventional method. To overcome the imbalance issue in transferring difficulty among classes, Zou et al. (2018) introduced class-balanced self-training by generating pseudo-labels with a balanced class distribution. Despite such progress, the abovementioned models often face challenges in real-world “wild tasks,” where large differences exist between labeled training/source data and unseen test/target data. UDA seeks to overcome this problem without using target-domain labels.

### 2.2. Semi-supervised learning with dual student

In consistency-based methods, the following two roles are commonly created, either explicitly or implicitly: a teacher model and a student model (i.e., a teacher–student structure). The teacher can be summarized as being generated by an exponential moving average (EMA) of the student. Ke et al. (2019) showed that these methods lead to a performance bottleneck as a coupled EMA teacher is not sufficient for the student. To overcome this type of problem, the model must learn the knowledge coming from another independent model instead of the EMA teacher. Ke et al. (2019) proposed to use dual student models to share the same network architecture with different initial states and to update them separately to avoid the limitation of the weight-coupling problem in the mean teacher. However, the outputs of the two models may differ significantly, and the direct application of the consistency constraint causes them to collapse with each other by exchanging incorrect knowledge. A stabilization constraint was thus proposed to overcome this problem, i.e., to define and obtain reliable knowledge of the models and exchange reliable knowledge with each other. Extensive experiments (Ke et al., 2019) have shown that this framework is effective, and it has yielded promising results when applied to the image-classification datasets CIFAR, SVHN, and ImageNet. Therefore, we employed this concept in our brain tumor segmentation and domain-adaptation experiments and achieved superior performance to those of UDA baselines and the conventional teacher–student structure.

### 2.3. Adversarial training

In the case of UDA, adversarial training is the most common and explored approach for semantic segmentation. The objective behind this is to adapt the segmentation network to be invariant to variations between the source and target. Zhang et al. (2017) proposed the use of both annotated and unannotated images in the segmentation pipeline and constructed two types of inputs for the evaluation network by using concatenation and element-wise multiplication. Luo et al. (2018) proposed adaptive weighting of the adversarial loss of different features, emphasizing the importance of category-level feature alignment for reducing domain shifts. Recent work on adversarial training for medical image segmentation indicates that the regulation effect of adversarial loss is applied to the internal features of the segmentor to achieve domain invariance, which is viewed as an adaptively learned similarity measure between the segmented outputs and the annotated ground truth. In this study, we computed the adversarial loss ($L_{\text{adv}}^{i}$) for every model and back-propagated it with the supervised loss and cross-coordination consistency loss to the segmentation network (G) for target image predictions. The discriminator (D) was trained with cross-entropy loss by using both domains and was designed to distinguish the domain of the input.

## 3. Methodology

**Figure 2:**
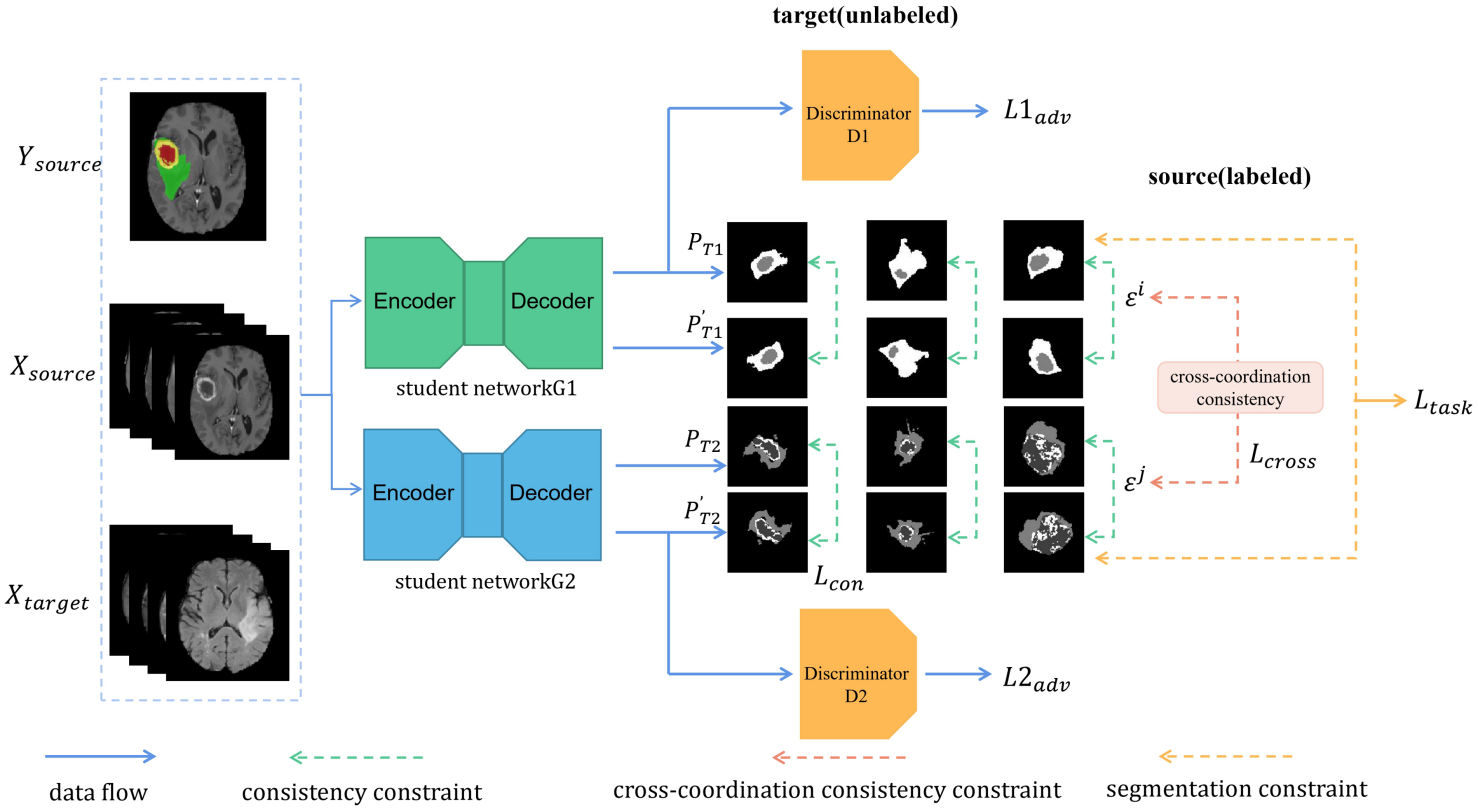
Illustration of our proposed architecture. Two models are trained independently. Each batch includes labeled data (from the source domain) and unlabeled data (from the target domain). As unlabeled data, we used the transformed segmentation maps generated with the original images as the input and the alternative segmentation maps generated with the transformed images as the input to meet the consistency constraint. In addition, the cross-coordination constraint is enforced between the students by their prediction and the perturbed one. Furthermore, each student must learn the segmentation task constraint for the labeled data.

In this section, we present our proposed framework for UDA on the BraTS 2019 dataset (Figure 2). Our model acts as a generator and is responsible for predicting segmentation maps for the input image, which could stem from the source or target domain, while the discriminator (D) takes the segmentation maps and predicts the domain of the input by means of an output of 1 or 0. The segmentation network attempts to fool the discriminator, which is achieved using a fully convolutional neural network, thus yielding features from the two domains with the same distributions. The combination of dual student and adversarial training allows both student models to have the ability to train and distinguish the domain of the input independently, without misleading each other. The cross-coordination consistency constraint allows both models to be interactive and learn from each other.

### 3.1. Proposed model

Our UDA framework consists of the two modules as shown in Figure 2: (i) the student networks G1 and G2 with different initializations and (ii) the discriminators D1 and D2, which are separately in charge of adversarial training and feature alignment for G1 and G2, respectively. First, we forward propagated the labeled images as the source domain and the unlabeled images as the target domain in a batch through each segmentation network (G1 and G2). Then, we updated the network weights through the ground truth from the source domain and obtained pre-softmax layer predictions about the unlabeled target domain. Predictions from both domains were passed through the discriminators D1 and D2 to distinguish whether the input belonged to the source or target domain. The networks G1 and G2 act as a generative model, and adversarial loss from D1 and D2 is back-propagated through the G1 and G2 networks, respectively, to update the network weights to learn domain-invariant feature representation. The predictions (P) of the original target-domain data from the networks G1 and G2 are transformed into $P_{T1}/P_{T2}$. The predictions (P′) of the transformed target-domain data from the networks G1 and G2 are noted as $P_{T1}^{\prime }/P_{T2}^{\prime }$. We computed the consistency loss between $P_{T1}/P_{T2}$ and $P_{T1}^{\prime }/P_{T2}^{\prime }$ and back-propagated the losses through the student networks G1 and G2. Finally, cross-coordination consistency was applied to the predictions from the target domain between the student networks G1 and G2 and was back-propagated through all of them.

### 3.2. Supervised source-domain adaptation

A robust model is the basis of source and target-domain training. Our models were trained with a supervised loss on the source domain. Formally, we set $X_{s}\subset {\mathbb{R}}^{H\times W\times D\times C}$ of the BraTS 2019 source examples along with associated ground truth C-class segmentation maps; $Y_{s}\subset {\left(1,C\right)}^{H\times W\times D}$ provides the label of pixel *(h, w, d)* as a one-hot vector. H, W, and D are the height, width, and depth, respectively, of the image and label. Let F be the segmentation network, which acquires an image x and predicts a C-dimensional “soft-segmentation map.” We chose dice loss and cross-entropy loss as our supervised segmentation loss $L_{\text{seg}},$ and the segmentation constraint can be computed as follows:


(1) $$L_{\text{seg}}=0.5*\left(L_{\text{dice}}+L_{ce}\right)\;\;\;$$

where


(2) $$L_{\text{dice}}=1-\frac{1}{M}\overset{M}{\underset{i=1}{\sum }}\left(\frac{2\sum_{x\in \Omega }p_{i}\left(x\right)\times y_{i}\left(x\right)}{\sum_{x\in \Omega }p_{i}\left(x\right)+\sum_{x\in \Omega }y_{i}\left(x\right)}\right)$$

(3) $$L_{ce}=-\frac{1}{M}\overset{M}{\underset{i=1}{\sum }}y_{i}\text{log}\left(p_{i}\right)$$

where $y_{i}$ and $p_{i}$ denote the ground truth and the probability that a pixel belongs to a category prediction, respectively, while M denotes the number of classes in the segmentation network G1/G2.

### 3.3. Dual student with adversarial learning

We combined adversarial learning with the dual student (Ke et al., 2019) to define the stable sample and stable constraint in our framework. To break the limit of the EMA model, we chose to initialize the weights of the networks by using the Xavier and Kaiming initializations and used the same training dataset for forward propagation by using U-Net (Ronneberger et al., 2015) and for back-propagation, respectively. Notably, we wished to emphasize the difference and independent learning of the two student models either in the way they are initiated or in the network architecture. In this manner, the student i weights $\theta^{i}$ were not an ensemble of the student j weights $\theta^{j}$ in a successive training step t with the smoothing coefficient α∈[0,1] like all existing teacher–student methods. Furthermore, if the student has biased predictions for specific samples, the EMA teacher is most likely to maintain the mistake learning and enforce the student to follow. A ramp-up operation for the consistency constraint is the most commonly applied one to alleviate this bias; however, this operation cannot solve the problem. In this case, training relatively independent but interactive models is beneficial as this gains loosely coupled targets. We used the dual student model as a regularizer to smoothen the weights of our feature space domain-adaptation network. The two student network weights were updated using task loss, cross-coordination consistency loss, and adversarial loss. For both our student models, we used the architecture proposed by Ronneberger et al. (2015).

### 3.4. Cross-coordination consistency constraint

If the outputs of the two student models vary widely, directly applying the consistency constraint will cause collapse due to the exchange of wrong knowledge. The EMA teacher does not suffer from this type of problem owing to the coupling effect. For good performance of the dual student, each of the student models must be able to reliably extract knowledge and effectively exchange knowledge with the other model.

As the BraTS 2019 dataset remains a significant challenge owing to the inclusion of multimodal data and domain-shift data, we put considerable effort into dealing with densely but inaccurately unlabeled target data. First, according to the smoothness assumption, a small perturbation must not affect the prediction of the samples. Therefore, we augmented the unlabeled images using operations such as random rotation/flip/reflection, contrast transformation, and noise perturbation. The transformed images were inputted into the two models to output the predictions $P_{T}{\;}_{i}$ and $P_{T}{\;}_{j}$. The original image predictions $P_{i},P_{j}$ of the two models were subjected to the same transformation to produce the newly transformed predictions $P_{ti}$, $\;P_{tj}$. If the transformed prediction $P_{T}{\;}_{i}$ of the original target data is in the classification prediction neighborhood of the prediction $P_{ti}$ of the transformed target data, this means that this sample has a high probability for the predicted label. The prediction consistency in its neighborhood can reflect the degree of stability of a sample x. Our dual adversarial structure also imposed the consistency constraint to meet the smoothness assumption and used the consistency loss $L_{\text{con}}$ for model training. For student i, the distance between $P_{T}{\;}_{i}$ and $P_{ti}$ was measured using the mean square error as follows:


(4) $$L_{\text{con}}^{i}=\frac{\sum {\left\|P_{T}{\;}_{i}-P_{ti}\right\|}_{2}}{n}$$

In addition to the abovementioned training details, to ensure that our dual adversarial student structure was trainable, we imposed the cross-coordination constraint on reliable samples from two independent models and introduced a Boolean function {*condition*}, followed by the method described by Ke et al. (2019) to measure the prediction consistency and indicate the reliability of *x*. For student *i*, the *condition* outputs one when it is true, and zero otherwise, as follows:


(5) $$R_{x}^{i}=\left\{P_{x}^{i}=P_{\bar{x}}^{i}\right\}\&\left(\left\{M_{x}^{i}>\xi \right\}\|\;\left\{M_{\bar{x}}^{i}>\xi \right\}\right)$$

where


(6) $$M_{x}^{i}=\;\;{\left\|f\left(\theta^{i},\;x\right)\right\|}_{\;\infty }$$

$\bar{x}$ is the noisy augmentation of a sample *x*. $P_{x}^{i}$ and $P_{\bar{x}}^{i}$ are the predicted labels of samples *x* and $\bar{x}$, respectively, by a student *i.* $M_{x}^{i}$ is the maximum prediction probability of model output. ξ∈[0,1] is a hyperparameter that indicates a confident threshold. If $M_{x}^{i}$ exceeds ξ, *x* is considered to be far from the decision boundary of the ground truth, i.e., this sample has a high probability for the predicted label. We also used the Euclidean distance to measure the prediction consistency as shown:


(7) $$\varepsilon_{x}^{i}=\;{\left\|f\left(\theta^{i},\;x\right)-f\left(\theta^{i},\;\bar{x}\right)\right\|}^{2}$$

Smaller distances indicate a more reliable *x*. In addition to measuring the reliability of *x* from one model, the distance measurement between the predictions of students *i* and *j* was key to calculating the cross-coordination constraint. Their distance was measured by $L_{MSE}$. Thus, the overall cross-coordination constraint for student *i* on sample *x* was written as follows:


(8) $$L_{\text{cross}}^{i}\left(x\right)=\left\{\begin{array}{c} \left\{\varepsilon_{x}^{i}>\varepsilon_{x}^{j}\right\}L_{MSE}\left(x\right),\;\;R_{x}^{i}=R_{x}^{j}=1\\ R_{x}^{j}L_{MSE}\left(x\right),\;\;\text{otherwise} \end{array}\right.$$

Notably, *x* stems from unlabeled target data and not from the training data. For student j, we calculated the cross-coordination constraint in the same manner and marked it as $L_{\text{cross}}^{j}$, which was as used in the training process.

### 3.5. Object function

With the proposed framework for training the student models *i* and *j*, we formulated the final loss function for the domain-adaptation task as follows:


(9) $$L=L_{\text{student}}^{i}+L_{\text{student}}^{j}$$

(10) $$L_{\text{student}}^{i}=L_{\text{seg}}^{i}\left(I_{s}\right)+\xi \left(L_{\text{con}}^{i}\left(I_{t}\right)+L_{\text{cross}}^{i}\left(I_{t}\right)\right)+\lambda_{\text{adv}}L_{\text{adv}}^{i}\left(I_{t}\right)$$

$L_{\text{student}}^{j}$ was calculated in the same way as $L_{\text{student}}^{i}$. $I_{s}$ and $I_{t}$ are inputs from the source and target domains, respectively. We used the averages of the dice and cross-entropy losses for the brain tumor segmentation task due to the low density and class imbalance issue of brain MR images. The consistency loss $L_{\text{con}}^{i}\left(I_{t}\right)$ was used to validate the predictions of each student model whether they were reliable or not. The cross-coordination constraint loss $L_{\text{cross}}^{i}\left(I_{t}\right)$ measures the difference in the predictions between the dual student model to avoid collapse and exchanging the wrong knowledge. The adversarial loss $L_{\text{adv}}^{i}\left(I_{t}\right)$ was calculated using the cross-entropy loss on unlabeled target predictions and labels to align the feature representations of the source and target domains. Because adversarial training may be less useful in the beginning stage of the training when the student model can produce good segmentation for the annotated training images, we set $\lambda_{\text{adv}}\;$= 0.1 initially and set $\lambda_{\text{adv}}\;$= 1 after all iterations. The value must be small (<1) when the student model can produce decent segmentation results. Furthermore, the discriminator networks were trained by the cross-entropy discriminator loss $L_{\text{disc}}\left(I_{s},I_{t}\right)$ using source and target feature representations.

### 3.6. Model architecture

*Segmentation network*: We used U-Net (Ronneberger et al., 2015) as our segmentation network with batch normalization, max pooling, and dropout. Networks were trained using stochastic gradient descent with momentum = 0.9; the weight decay was set to 1e − 4, and a poly learning rate decay policy was applied. The two student networks had identical U-Net architecture, and all student network weights were updated by back-propagation. The performance of the models was validated using target data and separately tested on holdout test sets.

*Discrimination network*: For discriminators, we used a fully convolutional neural network consisting of five convolutional layers with 4 × 4 × 4. kernels and a stride of 2. Except for the last convolutional layer, each convolutional layer was followed by a leaky rectified linear unit parameterized by 0.2 and a dropout probability of 0.5. Discriminators were trained with Adam as the optimizer with *β*_1_ = 0.45 and *β*_2_ = 0.999.

## 4. Experiments

### 4.1. Dataset and data split

We evaluated our proposed framework on the BraTS 2019 database (Menze et al., 2015). This dataset includes the images of 76 patients with LGGs and 259 patients with HGGs. All subjects were registered on different imaging modalities, such as T1-weighted, T1ce, T2-weighted, and FLAIR MRI in the preprocessing step. The BraTS 2019 dataset defines three labels on brain tumor images: peritumoral edema, enhanced tumor, and non-enhanced tumor. The source and target domains have the same classes. The whole tumor class includes all the abovementioned three labels; the tumor core class is a union of the enhanced and non-enhanced tumor labels, while the enhancing tumor core class is an independent class, which also constitutes a hyperactive part.

Following the procedure described by Liu et al. (2021), we evaluated our method according to the cross-subtype and cross-modality segmentation evaluation protocols.

For the HGG-to-LGG task, during the data preprocessing stage, we concatenated image slices from the four modalities as a four-channel input and resampled the images and paired labels to a spatial size of 128 × 128 × 128 to reduce the computational cost. Our training set included the labeled images of 259 HGG patients as the source-domain and the unlabeled images of 46 LGG patients as the target domain. The unlabeled images of the remaining 10 and 20 LGG patients were used as the validation and test sets, respectively.

For the cross-modality UDA task, we experimented on both the T2 + FLAIR to T1 + T1ce and T1 + T1ce to T2 + FLAIR tasks. For the T2 + FLAIR to T1 + T1ce task, we used two-channel images (from T2 and FLAIR) as the input and resampled them to a spatial size of 128 × 128 × 128 before the network training. We used the images of the HGG patients to test the performance of our framework. Our training set included the labeled T2-weighted + FLAIR MR images of 259 HGG patients as the source domain and the unlabeled T1 + T1ce MR images of 210 HGG patients as the target domain. The unlabeled T1 + T1ce MR images of the remaining 19 and 30 HGG patients were used as the validation and test sets, respectively.

For the T1 + T1ce to T2 + FLAIR task, the preprocessing steps were the same as the cross-modality task described earlier. Our training set contained the labeled T1 + T1ce MR images of 259 HGG patients as the source domain and the unlabeled T2 + FLAIR MR images of 210 HGG patients as the target domain. The unlabeled T2 + FLAIR MR images of the remaining 19 and 30 HGG patients were used as the validation and test sets, respectively.

### 4.2. Training protocol and evaluation metrics

#### 4.2.1. Experimental settings

For a fair comparison and analysis, we employed previous mainstream methods and our proposed framework to perform the same iterations with the same set of parameters for optimizers and learning rate decay. All training steps used a batch size of 2. We applied the poly learning rate, where the learning rate was multiplied by ${\left(1-\frac{\text{iter}}{\text{max\_iter}}\right)}^{\text{power}}$, where max_iter = 15,000 and power = 0.9. We also conducted extensive experiments on the BraTS 2019 dataset with the same dataset split and settings. The training was performed on a single NVIDIA RTX 3090 GPU with the PyTorch deep learning toolbox. We performed the following comparable experiments on the BraTS 2019 dataset:

(super-all): Training the segmentation network (with no domain adaptation) on the combined source and target data and validating and testing on the holdout target dataset.
(super-source): Training the segmentation network (with no domain adaptation) on source data alone and validating and testing on target data.
(da-mt): Domain adaptation using only mean teacher (Tarvainen and Valpola, 2017). Training the segmentation network on labeled source data and unlabeled target data and validating and testing on target data.
(da-entropy-mini): Domain adaptation using only entropy minimization (Vu et al., 2019).
(da-adv): Domain adaptation using only adversarial training (Zhang et al., 2017).
(da-ds): Domain adaptation using the dual student model (Ke et al., 2019).
(Ours): Proposed domain-adaptation framework using both dual student and adversarial training.

#### 4.2.2. Evaluation metrics

For the evaluation of the segmentation models, we adopted two metrics: the dice similarity coefficient (DSC) and Hausdorff distance (HD). The DSC measures the general overlap rate and the similarity between two sets of image data $\tilde{y}\;\text{and}\;y$, i.e., the similarity between sets of pixels. The DSC is the most widely used metric for the evaluation of image-segmentation models. It can be formulated as follows:


(11) $$DSC\left(\tilde{y},y\right)=\frac{2\times \left|\tilde{y}\cap y\right|}{\tilde{y}+y}$$

As a boundary-based metric, the HD is more sensitive than the DSC in terms of the segmentation boundary. The HD between two point sets is defined by the sum of all minimum distances from all points of a point set to another, divided by the number of points in a point set. The point sets represent our segmentation results and the ground truth, such that the maximum HD can indicate the maximum distance of the labeled and the predicted boundary.

### 4.3. Evaluation results

#### 4.3.1. Cross-subtype HGG-to-LGG UDA

**Figure 3:**
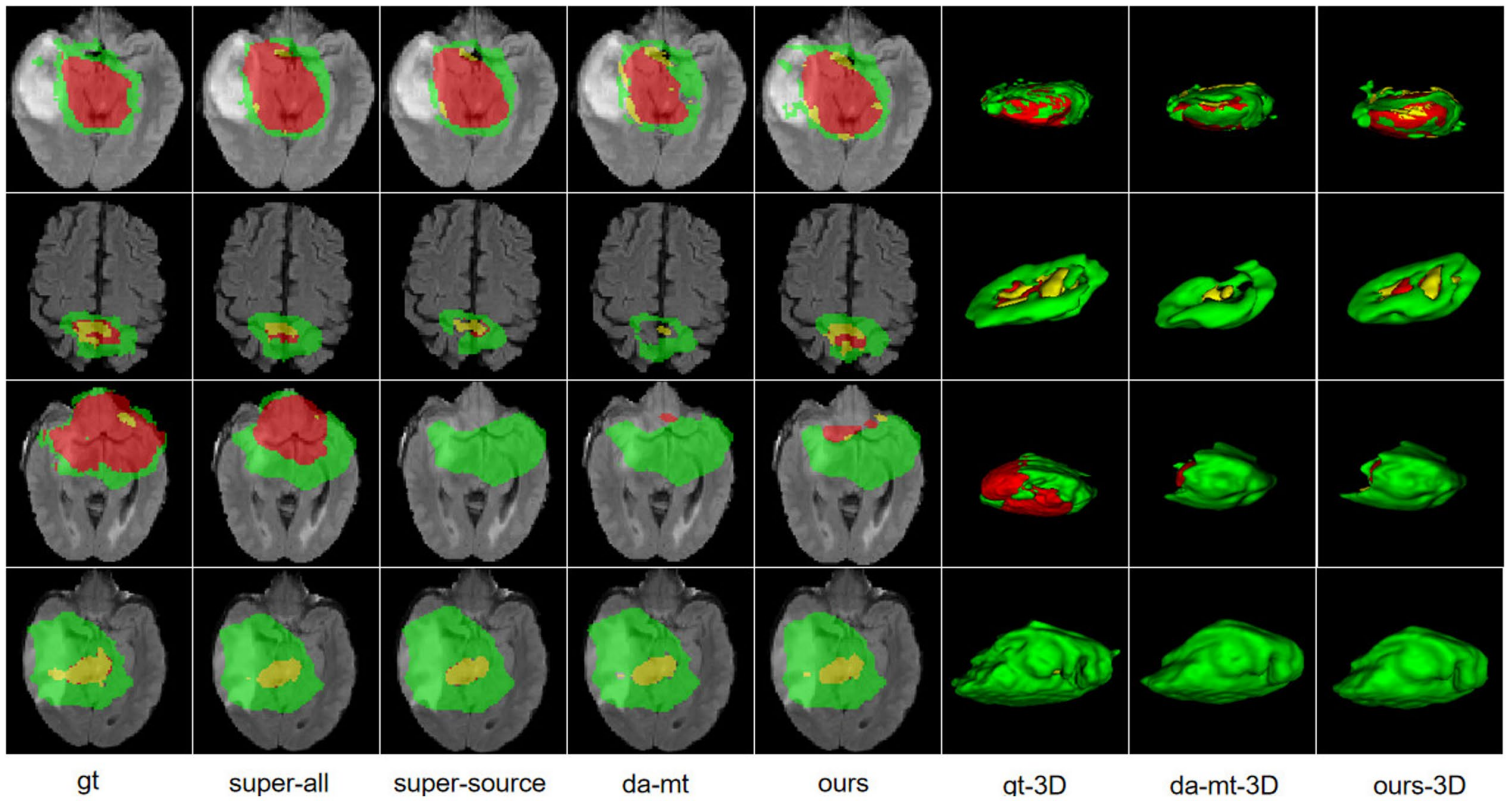
Illustration of the segmentation results of different unsupervised domain-adaptation methods in the high-grade glioma to low-grade glioma experiments. Yellow indicates the enhancing tumor core class. Yellow + red indicates the tumor core class. Yellow + red + green indicates the whole tumor class. gt, ground truth; da-mt; domain adaptation using mean teacher; ours, our framework.

HGGs and LGGs have different sizes and position distributions in terms of tumor regions. We trained all experiments for 15,000 iterations with HGG images as the source-domain and LGG images as the target domain. The networks were trained with four-channel 3D MRI volumes with a spatial size of 128 × 128 × 128 to perform four-class segmentation (background, enhanced tumor, whole tumor, and core tumor). The evaluation was implemented in the testing set consisting of LGG images. The performance scores for all experiments of the cross-subtype task are presented in Table 1. A comparison of the experimental results from the super-all and super-source models showed that the performance of the neural network models degraded drastically if the test data were from another domain than the training data. We attempted a different method, similar to the teacher–student structure and adversarial training, to complete the ablation study. In Table 1, the super-all model is the result of supervised learning and is used as a reference value. Our model is compared to the results of the super-all model for reference only. Compared to the super-source model, our model improved the dice scores of the whole tumor and enhanced tumor classes by 20.17 and 3.90%, respectively, and decreased the HDs of the whole tumor and core tumor classes by 2.13 mm and 1.04 mm, respectively. We compared the results of our model to the best experimental results, i.e., those of the da-adv model. We found that the HDs of the core tumor class were essentially the same for both models, decreasing by only 0.46 mm in our model. Thus, our proposed domain-adaptation method showed better performance overall, mitigated domain shift to an extent, and achieved noticeable improvement in segmenting the whole tumor and core tumor classes in the LGG dataset. The segmentation results are illustrated in Figure 3.

**Table 1:** Quantitative comparisons of DSC and HD in the HGG-to-LGG UDA task.

Experiment	DSC [%]↑			HD [mm]↓		
	WholeT	CoreT	EnhT	WholeT	CoreT	EnhT
Super-all	90.5062	64.9026	62.6651	8.19622	10.4381	10.1364
Super-source	68.6837	49.4666	59.7344	14.2450	15.0449	**9.4586**
da-mt	85.0194	27.1972	55.8531	13.9670	18.6337	14.0247
da-entropy-mini	85.5472	31.7202	54.8776	17.9886	18.0106	11.9540
da-adv	73.5623	45.4938	62.9094	14.3056	**13.5430**	10.2944
da-ds	84.0640	47.0138	**63.6998**	12.3527	14.0168	11.6854
Ours	**88.8554**	**48.3152**	63.6357	**12.1197**	14.0049	11.0748

DSC, dice similarity coefficient; HD, Hausdorff distance; HGG, high-grade glioma; LGG, low-grade glioma; UDA, unsupervised domain adaptation; WholeT, whole tumor; CoreT, core tumor; EnhT, enhanced tumor; da-mt, domain adaptation using mean teacher; da-entropy-mini, domain adaptation using entropy minimization; da-adv, domain adaptation using adversarial learning; da-ds, domain adaptation using dual student; ours, our framework. The bold numbers indicate the better experimental results for each segmentation target.

#### 4.3.2. Cross-modality T2 + FLAIR to T1 + T1ce and T1 + T1ce to T2 + FLAIR UDA

Multimodal images have abundant information that is effectively complemented, which improves the accuracy of segmentation but also increases its difficulty to a certain extent. Multimodal image information includes a large amount of unnecessary information, making the segmentation problem more difficult. We started our experiment using tumor image slices from different imaging modalities. To ensure the rigor of the controlled experiment, we used only the images of the HGG subjects for the experiment.

**Figure 4:**
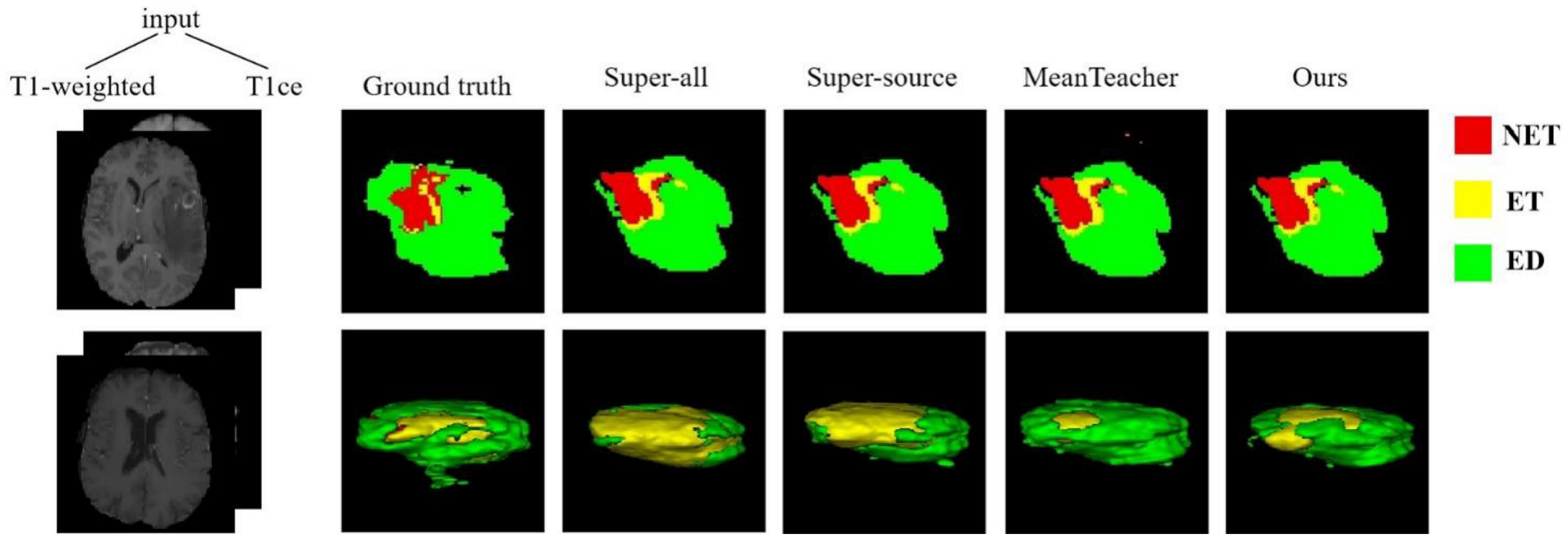
Examples of 2D slice and 3D segmentation results of different methods for T1-weighted and T1ce MR images in the T2 + FLAIR to T1 + T1ce UDA task. NET, non-enhancing tumor; ET, enhancing tumor; ED, peritumoral edema; T1ce, contrast-enhanced T1 images; FLAIR, fluid attenuated inversion recovery; UDA, unsupervised domain adaptation.

**Figure 5:**
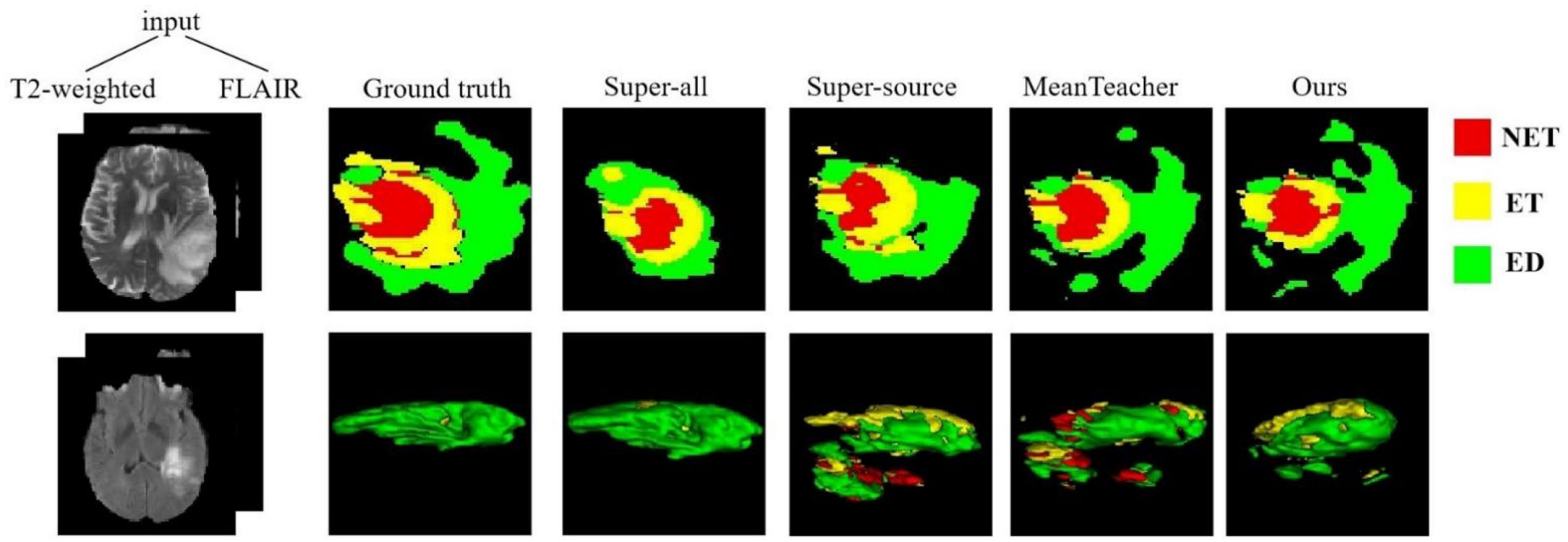
Examples of 2D slice and 3D segmentation results of different methods for T2-weighted and FLAIR MR images in the T1 + T1ce to T2 + FLAIR UDA task. T1ce, contrast-enhanced T1-weighted images; FLAIR, fluid attenuated inversion recovery; UDA, unsupervised domain adaptation; NET, non-enhancing tumor; ET, enhancing tumor; ED, peritumoral edema.

The quantitative evaluation results of the T2 + FLAIR to T1 + T1ce and T1 + T1ce to T2 + FLAIR tasks in the cross-modality UDA task are presented in Tables 2, 3, respectively. In addition to the quantitative evaluation, we visualized the segmentation results of different UDA methods for the two cross-modality UDA tasks (Figures 4, 5). As shown in Tables 2, 3, our proposed dual student with adversarial learning networks showed improved performance in the target domain and outperformed the mean teacher (da-mt) by a large margin but did not outperform the source model (super-source). We will continue to improve our network architecture in our future work.

**Table 2:** Quantitative comparisons of DSC and HD in the T2 + FLAIR to T1 + T1ce UDA task.

Experiment	DSC [%]↑			HD [mm]↓		
	WholeT	CoreT	EnhT	WholeT	CoreT	EnhT
Super-all	78.0329	85.8035	76.2321	19.0370	15.9223	16.1871
Super-source	33.9026	54.2546	40.5328	38.4868	32.4899	32.5497
Mean-teacher	27.8227	44.7233	35.4824	**41.9225**	**38.9808**	**39.0486**
Our method	**32.2734**	**45.7109**	**36.8492**	43.8065	41.5501	41.3097

DSC, dice similarity coefficient; HD, Hausdorff distance; T2, T2-weighted images; FLAIR, fluid attenuated inversion recovery; T1, T1-weighted images; T1ce, contrast-enhanced T1-weighted images; UDA, unsupervised domain adaptation; WholeT, whole tumor; CoreT, core tumor; EnhT, enhanced tumor. The bold numbers indicate the better experimental results for each segmentation target.

**Table 3:** Quantitative comparison of DSC and HD in the T1 + T1ce to T2 + FLAIR UDA task.

Experiment	DSC [%]↑			HD [mm]↓		
	WholeT	CoreT	EnhT	WholeT	CoreT	EnhT
Super-all	86.9167	59.1378	38.9827	18.8570	12.9443	12.7880
Super-source	58.3561	37.3261	23.5320	31.7271	33.6304	33.3393
Mean-teacher	30.2762	24.5957	15.7010	33.3301	34.6847	33.9832
Our method	**48.9012**	**31.2129**	**21.7738**	**31.2453**	**32.4403**	**32.6667**

DSC, dice similarity coefficient; HD, Hausdorff distance; T1, T1-weighted images; T1ce, contrast-enhanced T1-weighted images; T2, T2-weighted images; FLAIR, fluid attenuated inversion recovery; UDA, unsupervised domain adaptation; WholeT, whole tumor; CoreT, core tumor; EnhT, enhanced tumor. The bold numbers indicate the better experimental results for each segmentation target.

## 5. Discussion

We have presented a novel UDA framework based on semi-supervised methods for the tumor severity domain shift and cross-modality domain-adaptation tasks. Unlike other UDA methods that construct networks based on the characteristics of data from different domains, we did not work on designing the network but rather focused on establishing connections between the source-domain labels and the target-domain data. Despite the costly labeling, we had abundant biomedical images to utilize and could propose novel methods using source labels and the target-domain data to address UDA difficulties and alleviate the domain gap. Using a semi-supervised framework to solve the difficulties of UDA is a good research direction, and the purpose of our research in this study is also to try to utilize the advantages of semi-supervised approaches and achieve good performance on the BraTS 2019 dataset. Therefore, our framework has the potential to be applied to segmentation models that are stable in a source domain with the target-domain data from a range of clinical devices, in order to combat the problem of domain shift.

## 6. Conclusion

We have presented a novel approach to multi-modality and cross-modality domain adaptations by using the dual student model and adversarial training. We evaluated our model on the BraTS 2019 dataset, which has multimodal images and includes HGG and LGG patients, to address the cross-modality and tumor severity domain shifts. The results showed an improved segmentation performance in both tasks. The superior performance in both types of domain shifts validates the efficiency and accuracy of our proposed model. We plan to extend our method to other biomedical image-segmentation datasets to overcome domain-shift problems and improve the domain application. Future studies will include extensive hyperparameter tuning for medical image segmentation and UDA.

## Data availability statement

The raw data supporting the conclusions of this article will be made available by the authors, without undue reservation.

## Ethics statement

Ethics review and approval were not required for this study of images of human participants in accordance with the local legislation and institutional requirements. This research study was conducted retrospectively, using human subject data made available through the open-access dataset BraTS 2019.

## Author contributions

CQ, WL, and BZ designed the framework for brain tumor segmentation. BZ and JZ designed the experiments and analyzed the results. WZ and XZ analyzed the experimental datasets. CQ and WL were the major contributors to writing and editing the manuscript. JZ and CQ edited the manuscript. All authors contributed to the article and approved the submitted version.

## Funding

This study was supported by the NSFC (no. 61771347), the Special Project in Key Areas of Artificial Intelligence in Guangdong Universities (no. 2019KZDZX1017), the Guangdong Basic and Applied Basic Research Foundation (no. 2021A1515011576), the Basic Research and Applied Basic Research Key Project in General Colleges and Universities of Guangdong Province (no. 2021ZDZX1032), and the 2022 Guangdong Provincial Education Department Graduate Education Innovation Project (Guangdong Education and Research Letter [2022] no. 1).

## Conflict of interest

The authors declare that the research was conducted in the absence of any commercial or financial relationships that could be construed as a potential conflict of interest.

## Publisher’s note

All claims expressed in this article are solely those of the authors and do not necessarily represent those of their affiliated organizations, or those of the publisher, the editors and the reviewers. Any product that may be evaluated in this article, or claim that may be made by its manufacturer, is not guaranteed or endorsed by the publisher.
